# Correlation of Corneal Endothelial Cell Density with Corneal Tomographic Parameters in Eyes with Keratoconus

**DOI:** 10.4274/tjo.22800

**Published:** 2017-10-27

**Authors:** Banu Bozkurt, Mevlüt Yılmaz, Ali Meşen, Ümit Kamış, Bengü Ekinci Köktekir, Süleyman Okudan

**Affiliations:** 1 Selçuk University Faculty of Medicine, Department of Ophthalmology, Konya, Turkey; 2 Dr. Munif İslamoğlu State Hospital, Ophthalmology Clinic, Kastamonu, Turkey; 3 Dünyagöz Hospital, Ophthalmology Clinic, Konya, Turkey

**Keywords:** Keratoconus, cornea endothelial cell density, corneal tomography, specular microscopy, morphological changes

## Abstract

**Objectives:**

To examine changes in corneal endothelial cell density (ECD) in different stages of keratoconus and evaluate its correlation with corneal tomographic parameters.

**Materials and Methods:**

Two hundred six patients with keratoconus were enrolled in the study. Corneal topography was performed by Sirius (CSO, Italy), which has a rotating Scheimpflug camera and a Placido disc topographer. Automatic endothelial analysis was done with the non-contact endothelial microscope (20x probe) of Confoscan-4 (NIDEK, Japan). The eyes were classified into stages based on steepest keratometric value as follows: mild <45 D; moderate 45-52 D; severe >52 D and according to thinnest cornea thickness (TCT) as <400 μm, 400-450 μm, and >450 μm. Tomographic and endothelial cell parameters were compared among the groups using Kruskal-Wallis test and the correlations between them were analyzed using Spearman correlation.

**Results:**

The study included 391 eyes of 100 male (24.29±7.7 years, range 11-47 years) and 106 female (26.26±7.5 years, range 13-45 years) patients (p=0.07). Mean ECD values were 2628±262 cells/mm^2^, 2541.9±260.4 cells/mm^2^, and 2414.6±384.3 cells/mm^2^ in mild, moderate, and severe keratoconus, respectively (p<0.001) and 2592.3±277 cells/mm^2^, 2502±307 cells/mm^2^ and 2348±296 cells/mm^2^ in corneas with TCT values >450 µm, 400-450 µm, and <400 µm, respectively (p<0.001). ECD showed significant negative correlation with keratometric and elevation parameters and positive correlation with pachymetric parameters (p<0.05).

**Conclusion:**

As endothelial cell numbers seem to decrease with the progression of keratoconus, specular/confocal microscopy screening should be carried out, especially in eyes with advanced stages and corneas with TCT <400 µm.

## INTRODUCTION

Keratoconus is an ectatic corneal disorder characterized by progressive localized thinning and protrusion of the cornea, resulting in irregular astigmatism and decreased vision. Its annual incidence ranges between 50 and 230 per 100,000.^1^ Placido disc-based corneal topographies and measurement of corneal thickness are widely used methods in identifying cases of keratoconus. However, Placido-based corneal topographies evaluate only the anterior surface of the cornea and do not show corneal curvature and elevation of the posterior corneal surface.^2,3,4^ One of the most recent advances in corneal topography is the introduction of slit-scanning and Scheimpflug imaging systems, which take measurements both from anterior and posterior corneal surfaces. These systems provide more accurate, reliable, three-dimensional information about the shape of the cornea, including anterior and posterior corneal surface elevation data measurement and pachymetry map.^3,4,5,6,7^ Elevation measurements obtained by Orbscan and Pentacam have shown that corneal deformation also occurs in the posterior surface in eyes with keratoconus and that posterior elevation is the most sensitive parameter in discriminating keratoconus from normal corneas.^7,8,9,10,11^ Sirius (CSO, Italy) is a corneal tomography device which combines Scheimpflug photography analysis with the classic Placido disc technology. This provides highly consistent anterior and posterior corneal curvature measurements.^12,13,14^

Since keratoconus is an ectatic disease affecting both the anterior and posterior corneal surfaces, there might be changes in corneal endothelial cell number and morphology, especially in advanced stages of the disease. In keratoconus, evaluation of the corneal endothelium may be important since theoretically these cells may be damaged as a result of microscopic ruptures in Descemet’s membrane in ectatic areas, ultraviolet radiation damage due to stromal thinning, chronic eye rubbing, long-term contact lens wear, and oxidative stress.^15^ Keratoconus can also be associated with Fuchs’ corneal endothelial dystrophy.^16^ The status of cornea endothelial cells might alter the choice of keratoplasty technique (e.g. penetrating keratoplasty or Descemet stripping endothelial keratoplasty rather than deep anterior lamellar keratoplasty in eyes with low endothelial cell count) and is important in the decision to perform crosslinking (CXL) procedure or the selection of CXL protocols (transepithelial/hypotonic riboflavin solutions vs. isotonic dextran riboflavin solutions) to avoid endothelial cell toxicity in eyes with reduced endothelial cell count. However, our understanding of corneal endothelial changes in keratoconus remains incomplete. Histopathological evaluation of the corneal buttons of keratoconic eyes removed during penetrating keratoplasty has revealed deterioration in endothelial cell morphology and number.^17,18^ The findings of *in-vivo* confocal studies are conflicting.^19,20,21,22,23,24,25,26,27^ Specular microscopic examinations showed an increased variation in endothelial cell size (polymegathism) and shape (pleomorphism) in eyes with keratoconus.^28,29^ In a recent study, a trend toward lower endothelial cell density (ECD) and percentage of hexagonality, and a higher coefficient of variation was detected with advancing disease. However, there was no statistically significant correlation between the stage of keratoconus and changes in endothelial cell morphology and density.^30^ The conflicting results and the lack of significance in the correlation between ECD and topographic parameters might be due small number of keratoconus subjects within those studies.

In this study, our aim was to examine changes in corneal endothelial cells in different stages of keratoconus and evaluate the correlation of ECD with keratometric, pachymetric, and elevation parameters in eyes with keratoconus. Among studies evaluating the ECD and morphology in keratoconus, this study has the largest number of eyes with different stages of keratoconus.

## MATERIALS AND METHODS

The prospective cross-sectional study was performed in the Ophthalmology Department of Selçuk University Hospital. The study was approved by the Selçuk University Research Ethics Committee and followed the tenets of the Declaration of Helsinki. Written informed consent was obtained from all patients and the parents of those younger than 18 years.

Two hundred six patients with keratoconus were enrolled into the study. Each participant underwent a comprehensive ophthalmic examination including determination of uncorrected and best-corrected visual acuity, slit-lamp biomicroscopy, intraocular pressure measurement, and fundus examination. Eyes were diagnosed with keratoconus if they had at least one slit-lamp finding of anterior corneal bulging, stromal thinning, conical protrusion of the cornea at the apex, Fleischer ring, or Vogt striae and/or corneal topography findings characteristic of keratoconus such as asymmetric bow-tie pattern with or without skewed axes and inferior-superior power asymmetry. Exclusion criteria were past ocular surgery, contact lens wear, central corneal scarring, history of hydrops, or associated corneal dystrophies.

Corneal topography was performed using the Sirius corneal tomographer (CSO, Italy), which has a 360°-rotating Scheimpflug camera and a Placido disc topographer. Scheimpflug photography enables the acquisition and processing of 25 radial sections of the cornea and anterior chamber within a few seconds. Sirius takes measurements from 35,632 points for the anterior corneal surface and 30,000 for the posterior corneal surface in high-resolution mode in approximately 1 s or less and provides tangential and axial curvature data of the anterior and posterior corneal surfaces, refractive power of the cornea, and corneal pachymetry maps. A second camera checks that the alignment of the eye is maintained during measurement. Measurements were performed according to the manufacturer’s guidelines. The patients were seated in front of the machine and placed their chin on a chinrest and their forehead against the forehead strap. They were instructed to fixate on an internal fixation target and permitted to blink just before each measurement to spread an optically smooth tear film over the cornea and keep the eye open during image acquisition. Images with “OK” signal, which means that Scheimpflug acquisitions were above the required quality specifications for coverage and centration, were included in statistical analysis. Keratometry values in the flat (K1) and steep (K2) meridian, mean keratometry, thinnest corneal thickness (TCT), pachymetry at the apex of the cone (pachymetry apex), and highest anterior and posterior elevation values were recorded for each eye.

Measurements of corneal ECD and morphology were performed using the non-contact specular mode of Confoscan 4 (NIDEK, Japan). Non-contact endothelial microscope with 20x probe with a wider field of view was used for the measurement. The patient’s head was positioned similar to slit-lamp examination, and the patient was instructed to look straight ahead into the built-in fixation targets. Automatic focusing was used to ensure the image of the pupil on the monitor was in clear focus and within the aiming circle visible on the monitor. Three successive images were selected for the analysis. The central or paracentral area was determined by the operator and the automated cell analysis detected overall density, number of sides, and area of each cell as well as overall pleomorphism and polymegathism indices. Mean ECD, polymegatism, and pleomorphism of three images were calculated and recorded for statistical analysis.

Eyes were grouped into stages according to the collaborative longitudinal evaluation of keratoconus (CLEK) study group recommendation with respect to the curvature of the steepest corneal meridian (K2) as mild (<45 D), moderate (45-52 D), and severe (>52 D),31 and according to TCT as corneas less than 400 mm, 400-450 mm, and greater than 450 mm.

### Statistical Analysis

All statistical calculations were performed using SPSS (Statistical Package for the Social Science, version 16; SPSS Inc, Chicago, IL, USA) for Microsoft Windows. Data were statistically described in terms of mean ± SD and range. The normality of all data distributions was checked using the Shapiro-Wilk test. As the parameters were not normally distributed, tomographic and endothelial cell parameters were compared among the groups using Kruskall-Wallis test. In case of significance, Mann-Whitney-U test with Bonferroni’s adjustment for post-hoc analysis was used to analyze the differences between the two groups. The correlations between corneal tomographic parameters and endothelial cell parameters were analyzed using Spearman correlation. A p value <0.05 was accepted as statistically significant.

## RESULTS

The study included 391 eyes of 100 male (age: 24.29±7.7 years, range 11-47 years) and 106 female (age: 26.26±7.5 years, range 13-45 years), with no statistically significant differences in age or sex ratio (p=0.07).

According to the CLEK criteria, 39 eyes (10%) had mild keratoconus, 252 eyes (64.4%) had moderate, and 100 eyes (25.6%) had severe keratoconus. There were no significant differences in age among the 3 groups (p=0.07). Mean values of corneal tomographic parameters and endothelial cell parameters according to different stages of keratoconus are shown in Table 1. There was a statistically significant difference in ECD values according to stage of keratoconus, with the lowest value being in severe keratoconus (2628±262 cells/mm^2^, 2541.9±260.4 cells/mm^2^ and 2414.6±384.3 cells/mm^2^ in mild, moderate, and severe stages, respectively) (p<0.001).

There were 170 eyes with TCT >450 mm, 161 eyes with TCT between 400-450 mm, and 60 eyes with TCT <400 mm, with no statistically significant difference in respect to age (p=0.09) (Table 2). Mean values of corneal tomographic parameters and endothelial cell parameters according to TCT are presented in Table 2. Mean ECD values were 2592.3±277 cells/mm^2^, 2502±307 cells/mm^2^, and 2348±296 cells/mm^2^ in corneas with TCT values >450 µm, 400-450 µm, and <400 µm, respectively (p<0.001).

Overall pleomorphism and polymegathism indices did not differ among keratoconic eyes with different stages and thickness values (p>0.05) (Tables 2 and 3).

The correlations between ECD and keratometric values, anterior and posterior elevation parameters, and thickness parameters were statistically significant, but weak (r=0.17-0.26) (p<0.05) (Table 3).

## DISCUSSION

Despite the numerous studies in the literature evaluating corneal endothelial changes in keratoconus, there is still no consensus regarding whether endothelial cell count and morphology change in keratoconus and deteriorate with the progression of the disease. Among confocal studies which showed a decrease in ECD in keratoconus, Uçakhan et al.^19^ compared ECD in 48 eyes of 24 patients with keratoconus with 44 eyes of 22 healthy subjects and also among different stages of keratoconus using Confoscan 2.0 (NIDEK, Japan). Although they found lower ECD in keratoconic eyes (2754±312 cells/ mm^2^) than control eyes (2900±354 cells/mm^2^), this difference did not reach clinical significance. Mean ECD in eyes with severe keratoconus [mean K>55 diopter (D), n=26] was statistically significantly lower than in eyes with moderate (mean 47-55 D, n=17) (p<0.05) or mild (mean K<47 D, n=5) (p<0.05) keratoconus. The mean endothelial cell hexagonality percentage was statistically significantly lower in eyes with keratoconus compared to controls (p<0.05) and in eyes with severe keratoconus compared to mild or moderate keratoconus (p>0.05). Consistent with these findings, Mocan et al.^20^ found decreased endothelial cell count in eyes with keratoconus (2719±279 cells/mm^2^) compared to controls (2924±300 cells/mm^2^) with Confoscan 3.0 (NIDEK, Japan). In a study by Niederer et al.^21^ using laser scanning confocal microscopy (HRT, Heidelberg, Germany), ECD was found to be significantly reduced in eyes with keratoconus compared to controls (2412.2±339.5 cells/mm^2^ and 2845.6±313.0 cells/mm^2^, respectively), but the difference did not reach statistical significance between mild to moderate (steepest K<45 D and 45-52 D) keratoconus (21 eyes) and severe keratoconus (steepest K>52 D) (31 eyes) (2510.6±334.4 cells/mm^2^ and 2345.5±331.8 cells/mm^2^, respectively) (p=0.09). Bitirgen et al.^22^ found lower ECD in 78 keratoconic subjects with no history of contact lens use (2686±265 cells/mm^2^) compared to 36 age-matched control subjects (2875±223 cells/mm^2^) (p<0.001). El-Agha et al.^30^ evaluated the correlation between disease stage and corneal ECD and morphology in 40 eyes with keratoconus (11 eyes with stage 1, 17 eyes with stage 2, and 12 eyes with stage 3). They found lower ECD in eyes with stage 3 (2214.8±748 cells/mm^2^) compared to stage 1 (2404.5±345 cells/mm^2^) and stage 2 (2455.4±331 cells/mm^2^) (p=0.91). Advanced stage was also associated with higher coefficient of variation and lower percentage of hexagonal endothelial cells, although there was no statistically significant correlation. The lack of significant results may be explained by the small number of eyes within each group.

Some studies reported no endothelial changes associated with keratoconus.^21,22,23,24^ Using Confoscan, Weed et al.^23^ found no differences in ECD between keratoconus (n=19) (2888 cells/mm^2^ vs. 2941 cells/mm^2^ in moderate and severe cases, respectively) and healthy eyes (n=38) (3043 cells/mm^2^). Yeniad et al.24 used Confoscan 2.0 and observed no differences in ECD values among eyes with mild/moderate keratoconus and controls, even when the eyes were further subgrouped according to contact lens wear history. In a study by Timucin et al.^25^ no significant difference was found in ECD measured by laser scanning confocal microscopy in eyes with keratoconus (2731.6±303.2 cells/mm^2^) compared with controls (2664.9±319.5 cells/mm^2^) (p=0.4). They also could not show a significant difference according to disease stage when the eyes were classified as mild (steepest K<45 D, n=19), moderate (45-52 D, n=21), and severe (>52 D, n=25) (p=0.17). Ozgurhan et al.^26^ detected no differences in mean ECD among patients with manifest keratoconus (n=30), subclinical keratoconus (n=32), relatives of keratoconus patients, and a control group (p=0.592).

Interestingly, Hollingsworth et al.^27^ examined 29 keratoconus eyes and 29 age-matched healthy eyes using tandem scanning confocal microscopy (Tomey Confoscan) and showed increased endothelial cell count in eyes with keratoconus (3250±352 cells/mm^2^) compared to healthy eyes (3056±365 cells/mm^2^). The level of polymegathism did not differ between keratoconic subjects (0.35±0.05) and matched controls (0.38±0.07).

Conflicting results among studies might be due to inadequate number of eyes within the different stages of keratoconus, differences in exclusion criteria (current or previous contact lens wear), measurement devices (slit scanning or laser scanning confocal microscopy), techniques for image acquisition and cell density calculation (image size, image location, automated or manual cell counting), and classification of disease severity (Krumleich-Amsler, CLEK, etc). A central single assessment of less than 1/400 of the total number of endothelial cells in an eye with keratoconus may miss polymegathism, pleomorphism, and endothelial cell damage in the region of eccentric cone since the sample observed might not be representative.^15^ Furthermore, the accuracy of calculations decreases with lower image quality.

Most of the studies in the literature included a limited number of keratoconic eyes (less than 70), mostly in early and moderate stages, in which endothelial changes were not expected. Some studies showed decreased ECD in eyes with keratoconus but could not detect a statistically significant difference among the different stages of keratoconus, which might also be explained by small numbers of eyes within each stage. We think that a larger number of patients could have yielded statistically significant results in most of those studies. Therefore, we included a large number of keratoconic eyes (391 eyes) and classified eyes according to disease stage based on the CLEK recommendation and according to TCT. We used Sirius corneal tomographer (CSO, Italy), one of the latest corneal topography devices that combines Scheimpflug imaging with Placido-disc topography, and Confoscan-4 (Tokyo, Japan) with 20x probe, which images a wider field of view compared to other confocal systems and counts up to 1000 cells per exam. The automatic endothelial analysis gives the density plus polymegathism and pleomorphism indices, which makes Confoscan-4 a more objective device in the evaluation of corneal endothelium than other confocal microscopes. We found lower ECD in advanced stages of keratoconus and in thinner corneas, and the correlations between ECD and topographic parameters were significant (p<0.05). We excluded subjects who wear contact lenses or had a history of contact lens in the past, since wearing contact lenses was reported to cause a decrease in basal epithelial cell density, loss of keratocytes, and endothelial cell damage.^24,26,32^ In a study by Edmonds et al.^32^, after controlling for age and keratoconus severity, patients who wore SoftPerm contact lenses had 18% lower endothelial cell counts (2157±442 cells/mm^2^) than patients without contact lenses (2538±398 cells/mm^2^) and 15% lower than patients who wore soft toric disposable contact lenses (2483±292 cells/mm^2^). The large number of subjects, proper selection of cases, and use of the latest technologies make the results of our study more reliable and significant in terms of statistical analysis.

## CONCLUSION

In conclusion, as endothelial cell numbers seem to decrease with the progression of keratoconus, specular/confocal microscopy screening should be carried out, especially in eyes with advanced disease stage and corneas with TCT<400 µm.

## Figures and Tables

**Table 1:**
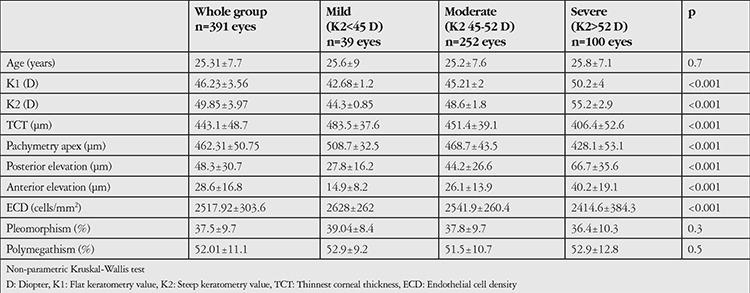
Corneal topographic and endothelial cell parameters according to keratoconus stage

**Table 2:**
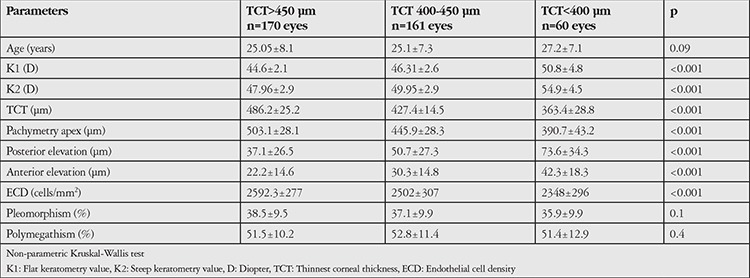
Corneal topographic and endothelial cell parameters according to thinnest cornea thickness

**Table 3:**
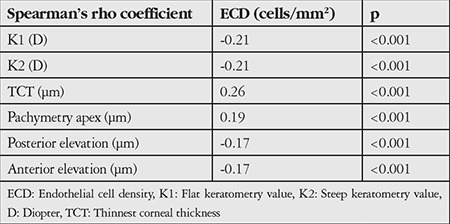
Correlations between endothelial cell density and corneal thickness and topographic parameters
